# Acceptability of Paper-Based Advance Care Planning (ACP) to Inform End-of-Life Care Provision for Community Dwelling Older Adults: A Qualitative Interview Study

**DOI:** 10.3390/geriatrics3040088

**Published:** 2018-12-05

**Authors:** Gary Bellamy, Jennifer Stock, Patricia Schofield

**Affiliations:** 1 Faculty of Health, Education, Medicine and Social Care, Anglia Ruskin University, Chelmsford CM1 1SQ, UK; patricia.schofield@anglia.ac.uk; 2 Department of Psychology, Institute of Psychiatry, Psychology & Neuroscience, King’s College London, De Crespigny Park, London SE5 8AF, UK; jennifer.stock@kcl.ac.uk; 3 South London and Maudsley NHS Foundation Trust, Bethlem Royal Hospital, Monks Orchard Road, Beckenham, Kent BR3 3BX, UK

**Keywords:** palliative and end-of-life care, older adults, advance care planning (ACP), health care professionals

## Abstract

This paper reports the findings from a study to investigate health care professionals’ views regarding the use and acceptability of two similar paper-based advance care planning (ACP) documents designed for older adults in their last year of life to inform end-of-life care provision. Participants’ views of using PEACE (Proactive Elderly Persons Advisory Care), a nurse led model with community geriatrician oversight, and PACe (proactive anticipatory care plan), a general practitioner (GP) led model implemented by two clinical commissioning groups (CCGs) as part of a wider pilot to determine their ability to improve end-of-life care provision, were explored. Hospital admission avoidance matrons took part in face to face interviews and care staff employed in private residential care homes took part in individual telephone interviews to explore their views of using the PEACE tool. Telephone interviews were conducted with GPs to explore their views of PACe. GPs and admission avoidance matrons were employed by CCGs and all study participants were recruited from the South East of England, where data collection took place in 2015. The data were analysed thematically. Findings from the study demonstrate how both tools provide a focus to ACP discussions to inform individual end-of-life care preferences. The importance of relationships was a pivotal theme established, trusting inter-professional relationships to enable multidisciplinary teamwork and a prior relationship with the older person (or their proxy in the case of cognitive impairment) to enable such conversations in the first place. Both tools enabled participants to think critically and reflect on their own practice. Notwithstanding participants’ views to improve their layout, using a paper-based approach to deliver streamlined ACP and end-of-life care was a theme to emerge as a potential barrier, and highlighted problems with accessing paper-based documentation, accuracy and care co-ordination in the context of multidisciplinary team working. The value of technology in overcoming this barrier and underpinning ACP as a means to help simplify service provision, promote integrated professional practice and provide seamless care, was put forward as a way forward.

## 1. Introduction

The UK population is ageing [1], with latest projections indicating an additional 5.5 million older adults in 20 years, increasing to approximately 19 million by 2050 (Ibid).

Whilst this is a cause for celebration, meeting the needs of older adults with long-term, often co-morbid, conditions as they approach the end of their lives in an equitable and timely manner remains a fundamental public health concern. In particular, end-of-life care for those living with dementia remains a critically important issue, both in terms of the high personal and social costs related to the disease, and the wider impact on other parts of the health and care system [2,3].

Older adults with a life-limiting illness should be offered the opportunity and support to discuss, review and document their individual care and treatment preferences at the end of life to achieve a ‘good death’. However, as Seymour et al. observe, historically, health and social care services have been slow to respond to the needs of older adults in their last year of life and their findings highlighted negative experiences [4]. Older people tend to receive poorer care towards the end-of-life compared to those who are younger, and are far less likely to be involved in discussions about their future care and treatment options. Moreover, they are less likely to die where they choose and less likely to receive specialist palliative care or access to hospice beds [5,6].

Dementia is now ranked in the top five underlying causes of death and current estimates suggest that one in three people who die after the age of 65 have will have the condition [7]. However, according to Hughes et al., the poor end-of-life care they experience can be either overly interventionist or suboptimal care, and complicated by the difficulty of identifying the end-of-life phase and difficulties in communication [8].

In 2008, the first “End-of-Life Care Strategy” for England and Wales indicated a key aim for services to meet the needs of people and that care is in keeping with any expressed preferences using advance care planning (ACP) [9]. 

ACP has been defined as ‘a voluntary process of discussion about future care and treatment preferences between an individual and their care providers, irrespective of discipline. This might include a discussion of the individual’s concerns and wishes, their important values or personal goals for care, their understanding of their illness and prognosis, and their preferences and wishes for the type of care or treatment that may be beneficial in the future and the availability of these [10] (p. 5). 

These discussions can result in the completion of written documents such as advance directives (including living wills and durable powers of attorney for health care) and do not attempt resuscitation (DNAR) orders, although there has been a shift to a more informal process of discussion and reflection about goals of care at the end-of-life [11,12,13]. This move was sanctioned by the Royal College of Physicians, who advise against the use of ACP being primarily document driven, or a “tick box” exercise [14]. Instead, the focus should be upon good communication, and any previous conversations highlighting care and treatment preferences should be subject to regular review and not performance measured on the completion of written documentation alone [15,16,17,18]. 

Developed from within a particular cultural and professional ideology which underscores the importance of individual autonomy, personal choice and a culture of practice promoting a particular vision of a ‘good death’, these discussions can enable end-of-life care provision that is in keeping with preferences of individual patients and family members [19]. They are important conversations that can change practice, inform and empower individuals to positively determine the manner of their own dying. These discussions can take place over a period of time, need not be too over-medicalised nor formalised, and can be undertaken by anyone involved in the provision of palliative and end-of-life care, although they are best undertaken by experienced staff following additional training [11].

The process of ACP can help facilitate patients’ future wishes regarding care and treatment preferences in the event of a loss of mental capacity [20], which can lead to less aggressive forms of medical care and better quality of life near death, decreased rates of hospital admission, especially those admitted from care homes, and increased rates of hospice admission. It can also be used as a basis for decision-making for those who retain the mental capacity, can enable a family to prepare, and can be used to resolve family conflict and deal with the subsequent bereavement whilst safe in the knowledge that the patients’ wishes have been carried out accordingly [21,22].

Health care professionals planning to engage in these conversations need to be cognisant of the risks and barriers that this particular age group have highlighted. Research indicates that some individuals may find the topic distressing and actively choose not to engage in discussions about the future to avoid thinking about deterioration in their condition [16,17,23]. Professionals cite the fear of jeopardising their relationship with patients [24], although being in a trusting relationship with the individual or having the ability to develop such a relationship is considered fundamental [25]. Skilled communication of healthcare professionals to facilitate these discussions is crucial [26], as well as collaboration between practitioners and family members, particularly in the case of cognitive impairment [27].

The UK Government’s June 2013 Spending Round announced the creation of a £3.8 billion Integration Transformation Fund (renamed the Better Care Fund), a pooled budget for health and social care services to work more closely [28]. This has been implemented in the context of an ageing population and those with long-term conditions to enable more effective integrated care [29,30]. One of the metrics for this fund relates to avoidable emergency hospital admissions. Older adults in their last year of life are likely to be admitted to hospital an average of 3.5 times [31] and the estimated cost of end-of-life care is billions of pounds [32]. Therefore, a multidisciplinary model of care with good communication between primary and secondary care providers is essential in end-of-life care to avoid unnecessary admissions and save costs [33]. Individuals and their families must be able to rely on safe, appropriate care, consistent with their expressed wishes, at any time of day or night, and regardless of the care provider. Timely ACP discussions and storing the documentation in one central place with shared access by health and social care providers could help with these challenges.

Other than lasting powers of attorney, the UK has no central registry for ACPs. Nonetheless, the 2008 UK National End-of-Life Care Strategy [8] recommended locality registers as a means to enable effective communication among professionals. The use of shared electronic health records to support the process of inter-agency working and provide seamless care has been highlighted in the Better Care Fund [29] as a means of providing coordinated, continuous care for patients and carers (see [34] as one such recent example). Frey et al. [35] suggest that technology supported information is fundamental to this and Bennett and Humphreys [29] advocate a high-touch, low-tech approach. Despite communication methods afforded by new technologies, sharing ACPs so that the appropriate services (hospitals, GP services and care homes) remain aware of updated plans remains a considerable challenge and service provision is variable. 

The concept of a ‘good death’ may be regarded as anathema—how could anything about dying be considered good?—but you only need to watch someone, or listen to the experiences of others who have witnessed someone die badly, to recognise that dying well is not only a valid goal but also hard to achieve. Whilst death and dying still remain largely taboo subjects, there remains mounting consensus in the public sphere both in the UK and internationally that talking about death and dying opens up the dialogue between older adults, their care professionals as well as their significant others, particularly in the case of cognitive impairment as a means to ensuring that end-of-life care provision is in keeping with personal preferences. Consequently, ACP is now regarded as the benchmark for good practice to promote discussion and avoid futile, costly and invasive treatments, enable death in the preferred place of care and avoid over-medicalising what is a natural phase of the life course.

With recent changes in funding arrangements, an ever increasing ageing population and the drive to streamline care, this paper provides the reader with an understanding of care professionals’ views regarding the use and acceptability of two similar paper-based advance care planning (ACP) documents to inform end-of-life care provision for community dwelling older adults, enabling them to spend their last days in familiar surroundings and reduce inappropriate hospital admissions [36].

## 2. Materials and Methods

The PEACE (Proactive Elderly Persons’ Advisory Care) ACP document was developed by Kings College Hospital, London in response to research and audits that highlighted how older adults, especially those with advanced dementia, were being admitted to hospital from care home facilities in the last days of life and dying in hospital in distress. PACe (Proactive Anticipatory Care plan) is a variation of the original PEACE plan. Four organisations piloting both documents for a 12 month period took part in the study: two NHS Clinical Commissioning Groups (CCGs) and two private residential aged care facilities located in the South East of England. Participants with experience using PEACE and PACe with older adults with end-of-life care needs were invited to participate via the commissioning leads in both CCGs responsible for delivery of the pilot. Their views in relation to using the documents were explored in individual interviews. Four care staff in two private residential homes and three care home admission avoidance matrons (*n* = 7) provided their experiences of using the PEACE tool—a nurse led ACP document with community geriatrician oversight. GPs (*n* = 5) gave an account of their experiences using the PACe ACP document (Proactive Anticipatory Care plan)—a GP led adaptation of PEACE for use across primary care, community and acute care settings. All study participants were highly qualified and experienced doctors, nurses and care workers with an in-depth knowledge of the individuals for whom they were providing palliative and end-of-life care. Ethical approval to conduct the study was obtained from the University of Greenwich Research Ethics Committee (approval reference FREC/EH/14-003, 2015). Study participants were invited to take part in either a short face-to-face or telephone interview with two university-based Research Fellows trained in qualitative methods (G.B. and J.S.). Prior to each interview, participants were provided with an information sheet and asked to sign a consent form indicating their willingness to take part in the study.

Telephone interviews were conducted with general practitioners (GPs). Three admission avoidance matrons took part in face-to-face interviews and four care staff employed by two residential care homes took part in individual telephone interviews. A total of nine telephone interviews and two face to face interviews (one joint and one individual) were held with twelve participants and lasted 15–30 min. A semi-structured interview guide (see Appendix A) was used as an aide-memoire and the interviews were digitally recorded and transcribed verbatim. Data collection took place between April–May 2015. Data analysis was conducted by G.B. and J.S. and adhered to the tenets of theoretical thematic analysis [37]. The method has been widely used across the social, behavioural and more applied (clinical, health, education) sciences. A six-phase approach was adopted which involved the following stages:

Familiarisation with the data: This phase involved reading and re-reading the verbatim interview transcripts, to become immersed and intimately familiar with their content.

Coding: This phase involved generating succinct labels that identified important features of the data.

Searching for themes: This phase involved examining the codes and collated data to identify significant broader patterns of meaning (potential themes).

Reviewing themes: This involved checking the candidate themes against the dataset, to determine that they told a convincing story of the data, and one that answered the research aims. In this phase, themes are typically refined, which sometimes involves them being split, combined, or discarded.

Defining and naming themes: This phase involved developing a detailed analysis of each theme, working out the scope and focus of each theme, and determining the story of each. It also involved deciding on an informative name for each theme.

Writing up: This final phase involved weaving together the analytic narrative and data extracts, and contextualising the analysis in relation to existing literature.

### 2.1. Results

Four major themes emerged from the data during data analysis: Existing relationships as a fundamental requisite to ACP discussions and documentation; inter-professional working relationships as a facilitator of ACP discussions and care delivery; reflective practice; and overcoming barriers with ACP documents to inform end-of-life care provision in line with personal preferences. Each of the four themes are discussed below and include verbatim excerpts from participants’ interviews.

#### 2.1.1. Existing Relationships as a Fundamental Requisite to ACP Discussions and Documentation

Rather than an expectation to engage in discussions and formulate plans without any prior medical knowledge of, or relationship with residents, patients, or their family members alike, participants maintained that knowledge of, and relationships with individuals were pivotal to the success of ACP discussions. In the context of dementia, where individuals were unable to engage in discussions about their own personal care and treatment preferences at the end-of-life, the advocacy role performed by staff and the views of family were fundamental in helping inform the plan of care. As one participant explains, the use of the relatives forum was crucial in seeking to establish the views of family members regarding the acceptability and views on the initiative, particularly where older adults themselves lacked capacity:

*“I’m quite lucky in that* (the care home manager) *will do quite a lot of the background work for me so she has forums with her relatives so she introduced at a forum where all of the relatives go and they all wanted all of their relatives to be on it* (PEACE plan)*.”*

(Admission Avoidance Matron. Female)

Participants expressed the opinion that interpersonal relationships were a fundamental requisite to end-of-life care discussions. As one participant goes on to explain, existing relationships with the patient, their family and being in a position of trust were all important factors:

“I think it is the clinician who has that relationship with the patient or their family in the case of somebody who doesn’t have capacity and somebody who they trust. I think it is the relationship that is important and I think I would say that the care staff would sort of have a better relationship with the family and see them, I mean I never see them, I’m just a GP that happens to phone them. I think it is more about the trust and relationship with that bit. I think sometimes it is, I don’t know, it’s difficult to explain to erm, yes I guess it is more about the relationship...”

(GP 1. Female)

In addition to the importance of prior knowledge of the patient, participants with experience of using the tools highlighted the importance of particular enablers for conversations to take place and the importance associated with good communication skills:

“...being able to say things in the right way, not being abrasive by saying ‘so how do you want to die or where do you want to die and clearly haven’t thought about it and clearly that’s not the best approach. I think a gentle way of leading the discussion towards...rather than just picking on the end of life, because there are all sorts of other avenues to discuss. And actually I think most people do respond very well to it so. I think its helpful doing it, know a patient, I think as the GP we know our patient and I think the nurse will know the patient well so they’ll already be in a position of trust. If you’d got somebody who didn’t know the person, that might not work and it’s not the most ideal approach.”

(GP 3. Female)

#### 2.1.2. Inter-Professional Working Relationships as a Facilitator of ACP Discussions and Care Delivery

Nursing and care home staff expressed the view of that working together and having the support from medical colleagues regarding advance care planning decisions and treatment options was fundamental to the success of both PEACE and PACe:

*“…I think it* (ACP) *works if everybody’s working together with it and has a clear understanding of what it is all about... the problem with a lot of these things is you start to do one thing with one person and they leave, unfortunately. It’s just everywhere, isn’t it? Someone else comes in who has a totally different idea or a totally different view on something and it means you’re kind of back to the beginning again, you know.”*

(Care Home Manager. Home B. Female)

Participants claimed that the support of a named member of the medical profession with whom they had a working relationship to sign off the PEACE plans gave them that extra credibility. As the following two interview excerpts demonstrate:

*“...so having their signatures behind that* (ACP document)*, having in-depth discussions with them* (medics) *adds a lot of weight...”*

(Admission Avoidance Matron. Female)

*“I think the credence lent to it* (PEACE) *by having game players if you like, like GPs and consultants, that lends a lot of credence to what is being discussed and it supports us which again gives it more credence to the kinds of discussions you are having and it makes you braver about having those discussions.”*

(Admission Avoidance Matron. Female)

Despite the majority of participants asserting the time consuming nature of completing ACP documentation, if they are done well, they felt that they saved time in the long run and served as the go to document for end-of-life care in keeping with individual preferences. In the case of care home staff, the documents provided them with the confidence to advocate on behalf of their care home residents—particularly night staff who often lacked the support that day staff could readily draw upon—as the following interview excerpts highlight:

*“They* (staff) *feel really thrilled with it* (PEACE tool)*. Especially the night staff. That’s why I brought that up because it was them that said it was nice that they’d got a plan as to what’s going to happen. We’ve always got lots of people that we can call in the day but at night and at the weekend you’re limited so they seem really pleased with it.”*

(Care Home Manager. Home A. Female)

*“Well, my own view is that it’s* (PEACE tool) *really helpful for us; we can see most of it that we can do for the resident in case we have some problems and then we can go to the PEACE plan and we will know their history and what we can do for them.”*

(Care Worker. Home A. Male)

### 2.2. Reflective Practice

Reflecting upon what the PEACE tool had afforded care home staff and residents alike, participants argued that it had strengthened their ability to advocate on behalf of their residents with end-of-life care needs and to afford them a good death in line with personal preferences:

*“I think it* (PEACE) *just reinforces what we are already doing. It’s very difficult because I don’t work on the floor as much as the others do but from my point of view I just feel it reinforces what we do and it also gives the senior carers peace of mind. They’ve got a document that they can refer back to as have I in times that I’ve thought ‘is there a PEACE plan for this’ and I’ve gone through it. I think it makes us think outside of the box more. I do think that rather than the knee jerk reaction now, I mean we’ve got one lady who’s not on the PEACE plan but it makes us think about PEACE...”*

(Care Home Manager. Home A. Female)

Participants also highlighted how the ACP documentation had enabled them to reflect on previous discussions with care home residents and family members and consider ways that potentially inappropriate hospital admissions could be avoided and anticipate issues that might arise in the future, as the following participant asserts:

*“I think some of it* (PEACE) *was helpful because it made us sit down and have discussions with residents and their families... the good thing about it* (PEACE) *was it was highlighting areas where you might have to send them back to hospital and areas where you could manage them within the home.”*

(Care Home Manager. Home B. Female)

In a similar manner, general practitioners with experience of using the tools highlighted how they had enabled them to reflect on their own practice, the conversations they had with patients in the last year of their life and use the lessons learnt to benefit future patients who were dying, as the following two excerpts demonstrate:

*“...it’s* (PACe) *definitely made me think more about you know, especially knowing sort of more about the next of kin and power of attorney and sort of thinking about that side of things as people, as sort of our elderly patients sort of just, I suppose as they deteriorate.”*

(GP4. Female)

*“It’s* (PACe) *made me think a little bit more, as a GP you tend to think a little bit deeper. It’s made me think a little bit more about the frailty issue, you know, on paper they look alright but looking at them....maybe it’s made me look at things a bit more laterally.”*

(GP 3. Female)

#### Overcoming Barriers with ACP Documents to Inform End-of-Life Care Provision in Line with Personal Preferences

Participants highlighted the problems that arose with using both documents in the initial stages when attempting to co-ordinate a meeting between health care professionals and family members to talk and plan care about a subject considered sensitive. As the following participant explains:

“It’s been really difficult to try to set up because you’re having to liaise at times when the family is available as well as myself so it has at times been a bit tricky and also we’ve had some conversations that have been a little bit tricky as well because you’re talking about end of life, you know, what we want, what’s going to be best and some people don’t like talking about these subjects and were also doing on how frail these residents are so it looks like whoever is going to deteriorate faster that’s who we put on the PEACE plan first. Like a priority order.”

(Care Home Manager. Home A. Female)

In their current, paper based format, participants highlighted the time consuming nature of collecting numerous signatures to prove that everyone involved in the plan had been consulted and was in full agreement with what had been documented:

“One of the difficult things I found was all the running and toing and froing, a lot of homes were very rapid, react as well as planned interventions. So it was very time consuming. You’d go, the families, the home, educating the home, getting all that on board, the patient themselves, then going to the GP who maybe wants something doing maybe slightly differently and then you went to the consultant so it’s a lot of man in the middle kind of thing.”

(Admission Avoidance Matron. Female)

The difficulties associated with the paper-based system were manifest in the accounts of those participants involved in using the PEACE and PACe tools respectively. They voiced the need for an electronic version to record and share information to improve its performance and interoperability and ensure the various care providers were ‘on the same page’ to provide consistent care:

*“Not to change the plan but I would have liked the fact that, it was the paperwork bit that got me...so I would meet with the relatives, I then had to go back, I had to bring it up on my computer back here, I then had to start writing it...if we’d had a laptop and I think this is something that we brought up, we could have done part of it as we went... It just felt laborious. You just think there’s got to be a way of speeding this up... but then part of me felt that would be rude but I’m not sure...or would it be better with a tablet? I don’t know but it just felt that part of why I was so slow was I could be writing it as I went and I did a lot of extra hours myself to make sure that I could say to the family, I will have it to you, and I kept thinking how much more can I keep doing...that we could just download it to streamline the process. It is the daunting factor that you’ve got to put the demographics in and it just felt like that and if there was just maybe a way to populate the patient’s details from system one, or whatever, because it was just a lot of me physically just putting in date of birth, all that kind of thing and you’re thinking...really?* (laughs)*.”*

(Admission Avoidance Matron’ Female)

Participants claimed that some of the difficulties that they experienced to make end-of-life care provision more seamless could be readily overcome if the tool could be shared electronically between the various care providers:

*“It* (PEACE) *needs to be electronic to make it a bit more seamless. That would help us in terms of, you could ping it securely to GPs, and it would help us and consultants by secure email.”*

(Admission Avoidance Matron. Female)

“Electronic, electronic, electronic. Let’s make it electronic basically!”

(Admission Avoidance Matron. Female)

## 3. Discussion

This study has provided an insight into the experiences, ideas and attitudes about the use of ACP discussions and documentation for older community dwelling adults with end-of-life care needs from a wide range of health care providers in England. The findings resonate with previous ACP-related research that points to the nature and importance of established, trusted relationships and the ability as a skilled communicator as prerequisites for health care practitioners to engage in discussions of this nature [25,26]. Moreover, the importance of collaborative relationships between different professional groups and the involvement of family members was evident in our findings to ensure end-of-life care provision is in line with personal preferences. In the context of dementia, this is consistent with the work of Ryan [2,27], who has suggested the development of close working relationships between practitioners and the families of people with dementia at the end of life.

Policy changes mentioned in this paper, particularly the End-of-Life Care Strategy [9] and more recently the Better Care Fund, demonstrate the impetus required to ensure provision of good end-of-life care for the UK’s ageing population. However, findings from this paper suggest the need to underpin care provision with a more streamlined, technological solution to ACP, to ensure personal preferences at the end-of-life phase are met. These findings are similar to concerns expressed by previous commentators [29,35]. A coordinated approach to care with access to interoperable IT systems are fundamental to the provision of equitable end-of-life care and ACP provision. Established data sharing agreements providing access to the latest centrally available ACP documentation are needed to circumvent any potential errors that may be associated with duplicate paper based systems to avoid unnecessary admissions into the acute setting. Community health and social care providers working together and caring for older adults are fundamental requirements to achieve individual preferences at the end-of-life. 

Technological development and innovation that keeps personal data security in mind, offers a fairly low-tech yet high-quality human touch, and prioritises the relationship between the older person or their proxy and care provider, is key to the delivery of compassionate care. An information technology approach to end-of-life care in old age does not have to be dystopian, but will require vigilance on the part of system developers, researchers, policy makers and providers alike to ensure that the creep of technology into end-of-life care for older adults is not just cheaper, but better and delivered with dignity.

## 4. Conclusions

Policy changes resulting from an increased ageing population have produced rapid changes to primary care provision in recent years, and local clinicians and local authorities have become empowered through the creation of clinical commissioning groups and health and wellbeing boards to co-ordinate health and social care and develop services aligned to local needs. This transformation in primary care as a result of the Better Care Fund is not without its own set of unique challenges for an increased ageing population. 

Older adults are more likely to be in receipt of treatment from different teams and organisations for existing, often co-morbid health conditions. Despite these challenges, they afford the timely opportunity to streamline ACP provision for older adults with palliative and end-of-life care needs living in the community towards achieving safe, personalised, proactive out-of-hospital and equitable end-of-life care. Whilst there is evidence of initiatives already in place that facilitate integrated ACP and end-of-life care as a means to achieve a ‘good death’, provision remains variable. This paper highlights the demand for similar ACP initiatives tailored to local needs, with the involvement of key stakeholders using a shared, integrated information technology platform. If equitable provision of end-of-life care for older adults is to become a reality, the resourcing of technology supported information concerning ACP delivery for health and social care providers to facilitate shared decision-making and information sharing is fundamental to effect the changes required.

Since death is now most likely to occur at the end of a very long life, community dwelling older adults living with dementia and diagnosed with palliative and end-of-life care needs are a growing group likely to derive considerable benefit from voluntary end-of-life care discussions to achieve a ‘good death’, adopting collaborative working approaches and underpinned by technological innovation.

## Appendix A

**PECE/PACe Interview Schedule**
- Introductions - Answer any questions - Explain the purpose of the interview - Sign consent form and complete demographic questionnaire

1. What are your views about the PEAC/PACe? 2. As the GP/nurse, do you feel you are the most appropriate person discuss/guide the future care and treatment preferences with patients/family members or should it be someone else? 3. Can you tell me a little bit about your experiences of using PEACE/PACe to date? 4. Has using the PEACE/PACe tool changed your practice? 5. Is there anything that you would do to change it? 6. Any further comments?
